# Modeling of Compressive Strength of Self-Compacting Rubberized Concrete Using Machine Learning

**DOI:** 10.3390/ma14154346

**Published:** 2021-08-03

**Authors:** Miljan Kovačević, Silva Lozančić, Emmanuel Karlo Nyarko, Marijana Hadzima-Nyarko

**Affiliations:** 1 Faculty of Technical Sciences, University of Pristina, Knjaza Milosa 7, 38220 Kosovska Mitrovica, Serbia; 2 Faculty of Civil Engineering, Josip Juraj Strossmayer University of Osijek, Vladimira Preloga 3, 31000 Osijek, Croatia; mhadzima@gfos.hr; 3 Faculty of Electrical Engineering, Computer Science and Information Technology, Josip Juraj Strossmayer University of Osijek, Kneza Trpimira 2B, 31000 Osijek, Croatia; karlo.nyarko@ferit.hr

**Keywords:** self-compacting rubberized concrete, compressive strength, machine learning, artificial neural networks, regression tree ensembles, support vector regression, Gaussian process regression

## Abstract

This paper gives a comprehensive overview of the state-of-the-art machine learning methods that can be used for estimating self-compacting rubberized concrete (SCRC) compressive strength, including multilayered perceptron artificial neural network (MLP-ANN), ensembles of MLP-ANNs, regression tree ensembles (random forests, boosted and bagged regression trees), support vector regression (SVR) and Gaussian process regression (GPR). As a basis for the development of the forecast model, a database was obtained from an experimental study containing a total of 166 samples of SCRC. Ensembles of MLP-ANNs showed the best performance in forecasting with a mean absolute error (MAE) of 2.81 MPa and Pearson’s linear correlation coefficient (R) of 0.96. The significantly simpler GPR model had almost the same accuracy criterion values as the most accurate model; furthermore, feature reduction is easy to combine with GPR using automatic relevance determination (ARD), leading to models with better performance and lower complexity.

## 1. Introduction

The European Union has prohibited all types of waste tire disposal since 2006 because the long process of tire deterioration affects the environment and wildlife. As natural resources are becoming increasingly scarce in the concrete industry, more emphasis is being placed on the utilization of waste products from other industries, such as the replacement of recycled aggregates with recycled rubber [1]. Waste rubber has a major impact on the properties of fresh concrete. The use of recycled rubber decreases the entry of aggressive substances into the material, guaranteeing that the concrete has less permeability and thus is more durable. It enhances impact, wear resistance and durability as well as other mechanical characteristics. It decreases compressive strength; shrinkage; and thermal conductivity coefficient, while increasing freezing resistance and sound absorption coefficient, depending on the amount and size of the rubber portion.

Self-compacting concrete (SCC) is utilized to enhance productivity, which means faster construction, reduced noise level, and improved surface finish, which eliminates the need for patching. SCC is a special type of concrete that does not need to use concrete compacting devices during casting [2,3]. Its self-buildability and self-leveling feature has eliminated the need of expensive vibrating equipment, reduced the cost of time of construction and the number of workers on site and increased safety at the site.

Unlike ordinary concrete, SCC contains a higher proportion of fine aggregate particles, and the water-binding ratio is lower, which affects the required force for the flow of concrete. Such a composition results in a decrease in the viscosity that is resolved by the addition of superplasticizers. Large amounts of recycled rubber particles in the material reduce the possibility of filling the formwork without additional vibration. In order to ensure the same properties or approximate properties of self-compacting rubberized concrete (SCRC) as in the reference mixture, it is necessary to modify and adjust the proportion of admixtures to the concrete. In this way, the concrete behavior with rubber in the fresh state is ensured. Even when the content of rubber particles is limited to a maximum of 20–30%, mechanical properties must be improved by adding higher content of cement and lower water to binder ratio or adding supplementary cementing materials, such as slag, silica fume or/and fly ash.

Moreover, the key indicator, commonly used for assessing the strength, the compressive strength of SCRC, generally decreases with the increase in content of rubber in SCRC. There are no expressions in building codes (for example, Eurocode 2 [4], or ACI Committee 209 [5]) for the prediction of compressive strength of rubberized concrete, especially of SCRC. Models from literature for predicting the compressive strength of rubberized concrete given by researchers are based on a reduction coefficient with respect to the referent mixture of concrete without recycled rubber particles. This implies that it is always necessary to make a reference mixture of concrete without the addition of crumb rubber particles. Therefore, in this article, an effort was made to model the compressive strength of SCRC by adopting one of several machine learning methods. So far, metaheuristic methods, and especially neural networks, have been successfully applied in various fields, such as in the control and optimization of processes, economics, medicine, and engineering [6,7,8,9,10]. They have also been used to model the properties of concrete in fresh or solid state [11,12,13,14,15], but much less in concrete with the addition of rubber [16,17,18].

Various researchers used different methods to model the compressive strength of rubberized concrete and SCRC. Some of the studies are summarized in Table 1. However, this field, especially when SCRC is in question, still requires further exploration.

Apart from ANN models, some other machine learning methods, such as k-nearest neighbor (KNN) and RF, have also been applied to estimate the compressive strength of concrete. KNN assigns unknown data values using the distances to the k-nearest data points. RF, on the other hand, is an ensemble learning method that generates a large number of decision trees during training. Unknown data values are assigned by RF based on the average prediction of the individual trees. For example, Ahmadi-Nedushan [30] developed a KNN model to predict the compressive strength of concrete with 104 experimental data, while Chopra et al. [31] estimated the compressive strength of concrete using an RF model with 49 data points.

The outcomes of the above studies are certainly encouraging, especially considering the fact that applications of ML models to estimate the compressive strength of SCRC are still at an early stage.

The aim of this article is to estimate the compressive strength of SCRC specimens using multilayered perceptron artificial neural network (MLP-ANN), ensembles of MLP-ANNs, regression tree ensembles (random forests, boosted and bagged regression trees), support vector regression (SVR) and Gaussian process regression (GPR). To the best knowledge of the authors, GPR with automatic relevance determination (ARD) has not been previously used for estimating SCRC compressive strength.

## 2. Methods

### 2.1. Multilayered Perceptron Artificial Neural Network (MLP-ANN)

Artificial neural networks are based on the parallel processing of various types of information similar to the human brain. They contain artificial neurons that are interconnected into a single parallel structure. A multilayer perceptron is a neural network with forward signal propagation that consists of at least three layers of neurons: input, hidden, and output layers.

**Figure 1:**
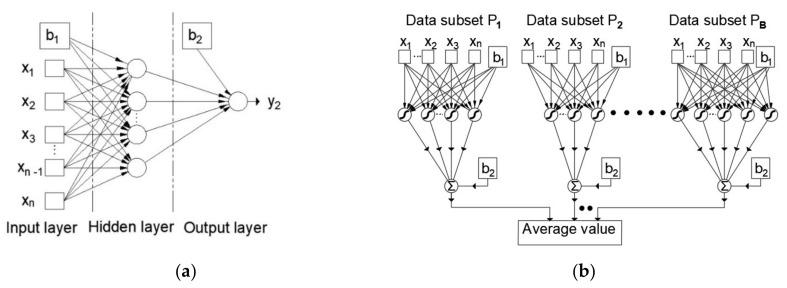
(**a**) Multilayer perceptron artificial neural network; (**b**) an ensemble of neural networks formed by the Bootstrap Aggregating (Bagging) approach [33,34].

In the general case, each neuron of one layer is connected to each neuron of the next layer, as shown in Figure 1 for the example of a three-layer MLP network with n inputs and one output. The properties of the network depend on the number of neurons and the type of activation function, so if the network is to be used as a universal approximator, it must use nonlinear activation functions in the hidden layer to be able to approximate nonlinear relationships between input and output variables [32]. A model with one hidden layer having neurons with a sigmoid activation function and output layer neurons with a linear activation function can approximate an arbitrary function when there is a sufficient number of neurons in the hidden layer [32].

As the number of input neurons is determined by the dimensions of the input vector and the number of output neurons by the dimension of the output vector, determining the network structure is reduced to determining the optimal number of hidden layer neurons if MLP architecture with the property of universal approximator is used.

The method for precise and reliable determination of the minimum required number of neurons has not been determined yet. What can be determined to some extent is the upper limit, i.e., the maximum number of hidden layer neurons, which can be used to model a system represented by a specific set of data. It is proposed to take into account a smaller amount of $N_{H}$ from the set of inequalities (1) and (2), where $N_{i}$ denotes the number of neural network inputs and $N_{s}$ denotes the number of training samples [33,34].

(1) $$N_{H}\leq 2\times N_{i}$$

(2) $$N_{H}\leq \frac{N_{s}}{N_{i}+1}$$

In order to improve the generalization of the model, when there is a small data with inherent noise, it is possible to train a larger number of neural networks and find the mean value of their outputs. In this way, ensemble models are created, while the individual models that make up the structure of the ensemble are called base models or submodels. The data set upon which the ensemble models were trained in each iteration is formed by the Bootstrap method [35]. The Bootstrap method forms a set of the same size as the original data set.

### 2.2. Regression Tree Ensembles

#### 2.2.1. Bagging

**Figure 2:**
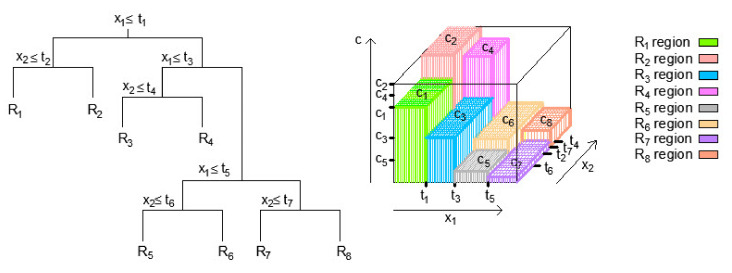
Space segmentation into regions and 3D regression surface in regression tree.

Methods based on classification trees (Classification and Regression Trees-CART) use the segmentation of the space of input variables in multidimensional rectangles or so-called boxes and then apply a model where multidimensional rectangles are assigned the appropriate value [35,36,37]. The lines that segment the input space are of the form $X_{i}=t$, with the remark that binary segmentation of the space is applied. Depending on the value of the input variables, the regression model (Figure 2) assigns a constant value of $c_{m}$ to each of the mentioned regions, which is equal to the mean value of the output variable for that region $R_{m}$, i.e., in this case:

(3) $$\hat{f}\left(X\right)=\overset{8}{\underset{m=1}{\sum }}c_{m}I\left\{\left(X_{1},X_{2}\right)\in R_{m}\right\}.$$

With the above procedure of forming a regression tree model, there is a possibility that the formed regression tree has good performance on the training set but poor generalization on the test data set. The Bootstrap aggregation-Bagging method allows for the aforementioned problem to be solved.

**Figure 3:**
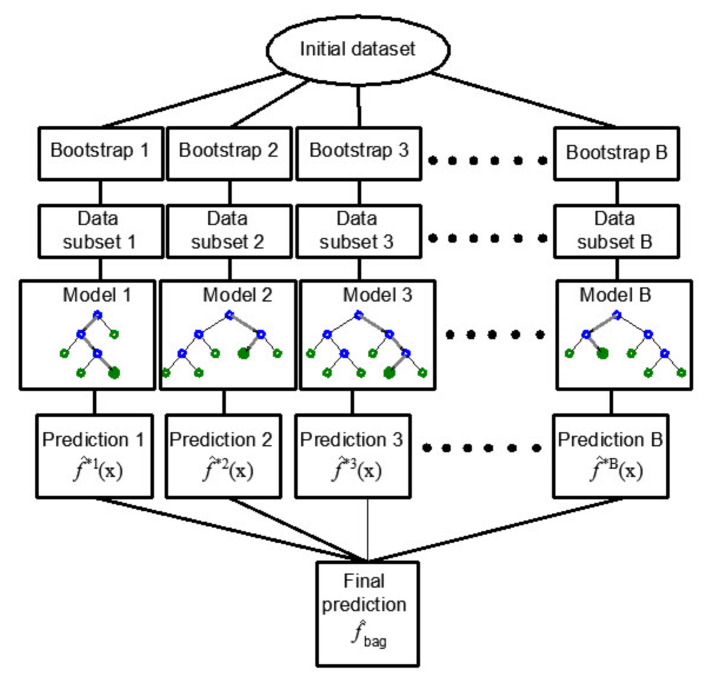
Bootstrap aggregation–Bagging in regression tree ensembles [34].

In order to practically carry out the mentioned procedure, it is necessary to have more training sets to reduce the variance by averaging. The mentioned problem of generating a more significant number of training sets can be overcome by the bootstrap method of sampling, i.e., by repeating sampling within the same training data set. The bagging method applies sampling with replacement [35]. If the model trained on the *b*-th bootstrap training set has the prediction function ${\hat{f}}^{*b}\left(x\right)$ at the point *x*, then by averaging all *B* models, a model (Figure 3) whose predictive function will be determined by the following expression can be obtained:

(4) $${\hat{f}}_{bag}\left(x\right)=\frac{1}{B}\overset{B}{\underset{b=1}{\sum }}\;{\hat{f}}^{*b}\left(x\right).$$

#### 2.2.2. Random Forests

The RF method differs from the Bagging method in that it does not use all the variables in generating the model. In the process of generating the ensemble, the method tries to form regression trees that are decorrelated, which leads to reduced variance when the ensemble results are averaged for all the trees within the ensemble [35].

Suppose that training dataset D is composed of l observations and n features. First, a sample from the training dataset is taken randomly with replacement and bootstrap is created. Before each split, m ≤ n  features are randomly selected as candidates for splitting. Typical values for m are approximately m/3 [35,38]. The RF model is obtained by aggregating individual tree models obtained in this way.

#### 2.2.3. Boosting Trees

The Boosting method uses sequential model training, where each new regression tree added to the ensemble has the function of improving the performance of the previous tree collection. In this part of the paper, the application of the Gradient Boosting method in regression trees will be discussed [35,39,40,41].

**Figure 4:**
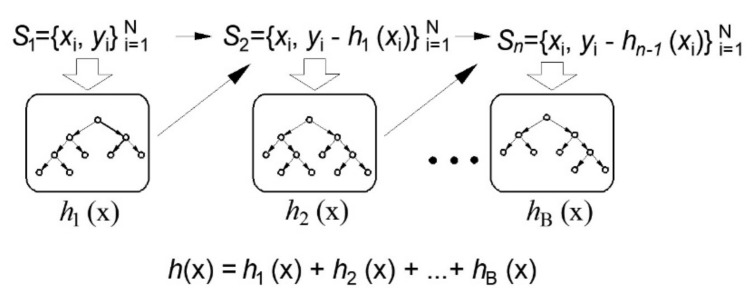
Gradient boosting in regression tree ensembles [34].

In the case of a quadratic error function (Figure 4), a new submodel is added to the basic model in each subsequent step, which best estimates the residuals of the previous model. In this way, by adding a model through the application of an iterative procedure, a definite model is obtained that represents an ensemble of previously obtained models.

Estimating the relative influence of the predictor variable in this method is based on the number of times a variable is selected for splitting, weighted by the squared improvement to the model as a result of each split, and averaged over all trees [42].

### 2.3. Support Vector Regression (SVR)

Suppose a training dataset $\left\{\left(x_{1},y_{1}\right),\;\left(x_{2},y_{2}\right),\ldots ,\;\left(x_{l},y_{l}\right)\right\}\in \;R^{n\;}\times R$ is given, where $x_{i\;}\in \;R^{n\;}$ is the n-dimenzional vector denoting the model’s inputs and $y_{i}$ are the observed responses to these inputs.

The approximation function has the following form:

(5) $$f\left(x\right)=\overset{l}{\underset{i=1}{\sum }}\left(\alpha_{i}{}^{*}-\alpha_{i}\right)K\left(x_{i},x\right)+b.$$

In Equation (5), K denotes the kernel function, and $\alpha_{i}$, $\alpha_{i}{}^{*}$ and b are the parameters obtained by minimizing the error function.

In order for SVR regression to be applied, the empirical risk function is introduced:

(6) $$R_{emp}^{\varepsilon }\left(w,b\right)=\frac{1}{l}\overset{l}{\underset{i=1}{\sum }}{\left|y_{i}-f\left(x_{i},w\right)\right|}_{\varepsilon .}\;$$

**Figure 5:**
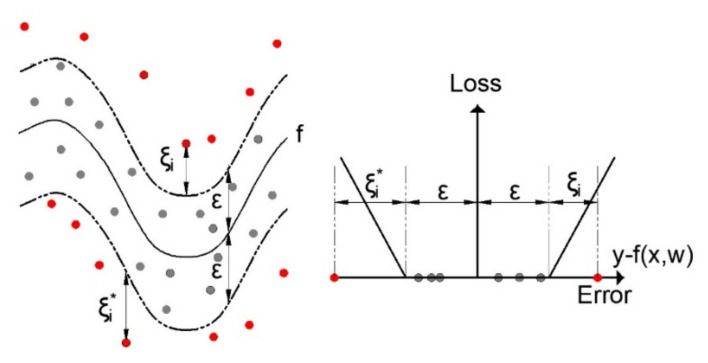
Nonlinear SVR with ε-insensitivity zone.

With the SVR algorithm, the goal is to minimize the empirical risk $R_{emp}^{\varepsilon }$ as well as the $w^{2}$ value simultaneously. The so-called Vapnik’s linear loss function (Figure 5) with ε-insensitivity zone is introduced, defined by the following expression [43,44]:

(7) y−fx,wε=0       if         y−fx,w≤ε  y−fx,w−ε    otherwise.

Considering the above expression, the problem can be reduced to minimizing the following function:

(8) $$R=\frac{1}{2}{||w||}^{2}+C\overset{l}{\underset{i=1}{\sum }}{\left|y_{i}-f\left(x_{i},w\right)\right|}_{\varepsilon .}\;$$

The constant C has the role of balancing between the approximation error and the norm of the weight vector w. Minimizing R is equivalent to minimizing:

(9) $$R_{w,\xi ,\xi^{*}}=\frac{1}{2}\left[{||w||}^{2}+C\left(\overset{l}{\underset{i=1}{\sum }}\xi +\overset{l}{\underset{i=1}{\sum }}\xi_{*}\;\right)\right],$$

where ξ and $\xi^{*}$ are the slack variables, which are shown in Figure 5.

Linear, RBF and sigmoid kernels used in this paper are defined as [45]:

(10) $$Kx_{i},x=x_{i},x$$

(11) $$Kx_{i},x=\exp\left(-\gamma x_{i}-x^{2}\right),\;\gamma >0$$

(12) $$Kx_{i},x=\tanh\left(\gamma x_{i},x+r\right),\;\gamma >0$$

In this paper, LIBSVM software with SMO optimization algorithm was used [46,47]. The LIBSVM software was used within the MATLAB program [47].

### 2.4. Gaussian Proces Regression

A Gaussian process model is a probability distribution over possible functions that fit a set of points. Consider a problem of nonlinear regression:

(13) $$y=f\left(x\right)+\varepsilon ,\;\;\;\;\varepsilon \sim Ν\left(0,\;\sigma^{2}\right).$$

where the function $f\left(\cdot \right)\;:\;R^{n}\to R$ is unknown and needs to be estimated, $y_{i}$ is target variable, x are input variables and ε is normaly distributed additive noise. Gaussian process regression [48] assumes that $f\left(\cdot \right)$ follows a Gaussian distribution with mean function $\mu \left(\cdot \right)$ and covariance function $k\left(\cdot \;,\;\cdot \right)$. The n observations in an arbitrary data set $y=\left\{y_{1},\ldots ,y_{n}\right\}$ can always be imagined as a sample from some multivariate (n variate) Gaussian distribution:

(14) $${\left(y_{1},\ldots ,y_{n}\right)}^{T}\sim N\left(\mu ,\;Κ\right),$$

where $\mu =\left(\mu (x_{1}\right),\ldots ,\mu (x_{n}){)}^{T}$ is the mean vector and *K* is n×n covariance matrix of which the $\left(i,j\right)$ th element $K_{ij}=k\left(x_{i},\;x_{j}\right)+\sigma^{2}\delta_{ij}$. Here, $\delta_{ij}$ is Kronecker delta function. Let $x^{*}$ be any test point and $y^{*}$ be corresponding response value. The joint distribution of $\left(y_{1}\ldots ,y_{n},\;y^{*}\right)$ is an (n + 1) variate normal distribution $\left(y_{1},\ldots ,y_{n},\;y^{*}\right)\sim N\left(\mu^{*},\sum \right)$, where $\mu^{*}=\left(\mu (x_{1}\right),\ldots ,\mu (x_{n}),\;\mu \left(x^{*}\right){)}^{T}$ and covariance matrix:

(15) $$\sum =\left[\begin{array}{ccccc} K_{11} & K_{12} & \cdot \cdot \cdot & K_{1n} & K_{1*}\\ K_{21} & K_{22} & \cdot \cdot \cdot & K_{2n} & K_{2*}\\ \cdot \cdot \cdot & \cdot \cdot \cdot & \cdot \cdot \cdot & \cdot \cdot \cdot & \cdot \cdot \cdot \\ K_{n1} & K_{n2} & \cdot \cdot \cdot & K_{nn} & K_{n*}\\ K_{*1} & K_{*2} & \cdot \cdot \cdot & K_{*n} & K_{**} \end{array}\right]=\left[\begin{array}{cc} K & K^{*}\\ \;K^{*T} & K^{**} \end{array}\right]$$

where $K^{*}={\left(K\left(x^{*},x_{1}\right),\ldots ,\;K\left(x^{*},x_{n}\right)\right)}^{T}$ and $K^{**}=K\left(x^{*},x^{*}\right)$.

The conditional distribution of $y^{*}$, given $y={\left(y_{1},\ldots ,y_{n}\right)}^{T}$ is then $N\left({\hat{y}}^{*},\;{\hat{\sigma }}^{*}{}^{2}\right)$ with

(16) $${\hat{y}}^{*}=\mu \left(x^{*}\right)+K^{*T}\;K^{-1}\left(y-\mu \right),$$

(17) $${\hat{\sigma }}^{*}{}^{2}=K^{**}+\sigma^{2}-K^{*T}\;K^{-1}K^{*}.$$

For some covariance functions, hyperparameters can be used to determine which inputs (variables) are more relevant than the others, using the automatic relevance determination (ARD). For example, consider the squared exponential covariance function with different length scale parameters for each input (ARD SE):

(18) $$k\left(x_{p},\;x_{q}\right)=v^{2}exp\left[-\frac{1}{2}\overset{n}{\underset{i=1}{\sum }}{\left(\frac{x_{p}^{i}-x_{q}^{i}}{r_{i}}\right)}^{2}\right].$$

where $r_{i}$ denotes the length scale of the covariance function along the input dimension i. If $r_{i}$ is very large, the relative importance of the i-th input is smaller [48]. The hyperparameters $\left\{v,r_{1},\ldots ,r_{n}\right\}$ and the noise variance $\sigma^{2}$ can be estimated by the maximum likelihood method. The log-likelihood of the training data is given by:

(19) $$L\left(v,r_{1},\ldots ,r_{n},\sigma^{2}\right)=-\frac{1}{2}\log\det\;K-\frac{1}{2}\;y^{T}\;K^{-1}y-\frac{n}{2}\log2\pi .$$

## 3. Evaluation and Performance Measures

**Figure 6:**
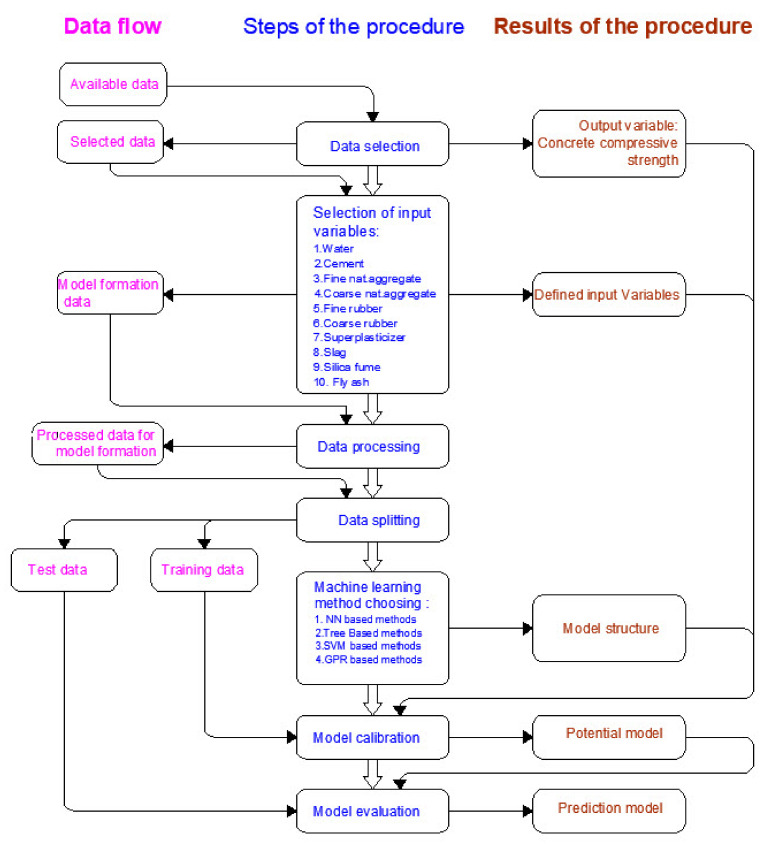
Systematic approach in forming prediction models.

The aim of defining the procedure for forming a model is to divide the whole process of forming a model into a certain number of steps (Figure 6) so that each time a model is formed, the same procedure is applied and all models are formed under the same conditions. The same criteria for accuracy assessment were defined and applied to the models.

The root mean square error (RMSE), mean absolute error (MAE), Pearson’s Linear Correlation Coefficient (R) and mean absolute percentage error (MAPE) were used to assess the quality of the model.

The RMSE criterion for evaluating the accuracy of a model is a measure of the general accuracy of the model and is expressed in the same units as the quantity to be modeled:

(20) $$RMSE=\sqrt{\frac{1}{N}\overset{N}{\underset{k=1}{\sum }}{\left(d_{k}-o_{k\;}\right)}^{2},}$$

where:

$d_{k}$—actual value (target value),

$o_{k}$—output or forecast given by the model,

*N*—number of training samples.

The *MAE* criterion is a measure of the absolute accuracy of the model and is used to represent the mean absolute error of the model:

(21) $$MAE=\frac{1}{N}\overset{N}{\underset{k=1}{\sum }}\left|d_{k}-o_{k\;}\right|$$

Pearson’s linear correlation coefficient *R* represents a relative criterion for evaluating the accuracy of the model:

(22) $$R=\sqrt{{\left[\overset{N}{\underset{k=1}{\sum }}(d_{k}-\bar{d})\left(o_{k}-\bar{o}\right)\right]}^{2}\times {\left[\overset{N}{\underset{k=1}{\sum }}{\left(d_{k}-\bar{d}\right)}^{2}{\left(o_{k}-\bar{o}\right)}^{2}\right]}^{-1}}$$

where $\bar{o}$ represents the mean value of the prediction obtained by the corresponding model and $\bar{d}$ represents the mean target value. Correlation coefficient values greater than 0.75 indicate a good correlation between the variables [49].

The mean absolute percentage error (*MAPE*), defined by Equation (23), represents a relative criterion for evaluating the accuracy of the model,

(23) $$MAPE=\frac{100}{N}\overset{N}{\underset{k=1}{\sum }}\left|\frac{d_{k}-o_{k\;}}{d_{k}}\right|$$

The paper uses the procedure with ten-fold cross-validation of the model.

## 4. Dataset

A systematic search for papers examining the properties of SCRC in fresh and solid state was performed in April 2020.

Based on papers published regarding the modelling of compressive strength of self-compacting rubberized concrete, input parameters were selected (Table 2) and all data that were incomplete or that did not include any of the selected input parameters were removed from the database.

With the aim of improving the mechanical properties of SCRC, various supplementary cementing materials, such as slag, silica fume or/and fly ash, which are used in concrete mixtures, have also been reported in the literature. Therefore, the SCRC mixtures containing supplementary cementing material, such as slag, silica fume and fly ash, are included in the database. The data collected by searching through research papers contain the results of 166 SCRC samples (Table 3). An overview of some of the data sources is provided as follows.

Emiroglu et al. [50] provided experiments with the aim of investigating the bonding performances of crumb rubber and reinforced bars in SCRC. The authors prepared four different R-SCC mixtures with the replacement of crumb rubber by volume with the natural aggregate in percentages of 15%, 30%, 45% and 60%. In the database, only mixtures with 15% of crumb rubber replacement were considered.

In order to investigate durability properties, Yung et al. [51] replaced part of the fine aggregate with waste tire rubber powder in volume ratios of 5%, 10%, 15% and 20%. They concluded that the best level of replacement achieved was with the addition of 5% waste tire rubber powder (that had been passed through a #50 sieve).

The results obtained by Li et al. [52] indicated that an SCC with adequate workability can be successfully created with partial replacement of sand or coarse aggregate with rubber particles of the same volume. When the replacement rate of sand with rubber particles was 30%, the value of loss of compressive strength of SCC was about 30%. Therefore, this mixture with 30% replacement rate was not considered.

Khalil et al. [53] prepared SCC specimens with different ratios of crumb rubber (10%, 20%, 30% and 40% of volume replacement of sand), but the last two mixtures (with 30% and 40% of sand replacement) were not added in the database.

Yu [54] conducted a study on the effect of changing regularity of waste rubber on deformation performance of SCRC. The results showed that the rubber particles in a more uniform distribution reduced the maximum compressive strength.

Zaoiai et al. [55] compared the rheological and mechanical performance between different mixtures formulations in order to obtain the optimum dose for rubber particles. The results of experimental testing showed that the compressive strength of SCC slightly decreased by replacing natural aggregate with rubber granulates.

Ismail and Hassan [56] investigated the mechanical properties and impact resistance of SCRC mixtures in which steel fibers were added in order to reinforce SCRC. However, all mixtures with steel fibers were removed from the database. The results showed that the addition of crumb rubber to concrete improved impact energy absorption and ductility, while the mechanical properties decreased with increasing content of crumb rubber.

## 5. Results and Discussion

MLP-ANN with one hidden layer was trained using Levenberg-Marquardt algorithm [71]. The criterion to stop the training was either the maximum number of epochs (set to 1000), the minimum gradient magnitude (set to 10^−5^) or the network performance (measured as the mean square error and set to 0). All input data were normalized in the range [−1, 1] prior to training. The following variables are defined as input variables of the model: water, cement, fine natural aggregate, coarse natural aggregate, fine rubber, coarse rubber, superplasticizer, slag, silica fume and fly ash, and these input variables determine the number of neurons in the input layer (i.e., the variable water corresponds to the first neuron, the variable cement the second neuron of the input layer, etc.) of the ANN model. There are ten input variables, and the number of input layer neurons is ten. The number of neurons in the output layer in the regression problem is one, and the output of this neuron corresponds to the prediction of the compressive strength of concrete. The sigmoid activation function is used in the hidden layer, while the linear activation function is used in the output layer.

The maximum number of neurons in the hidden layer was determined experimentally using Equations (1) and (2) and equals 16.

**Figure 7:**
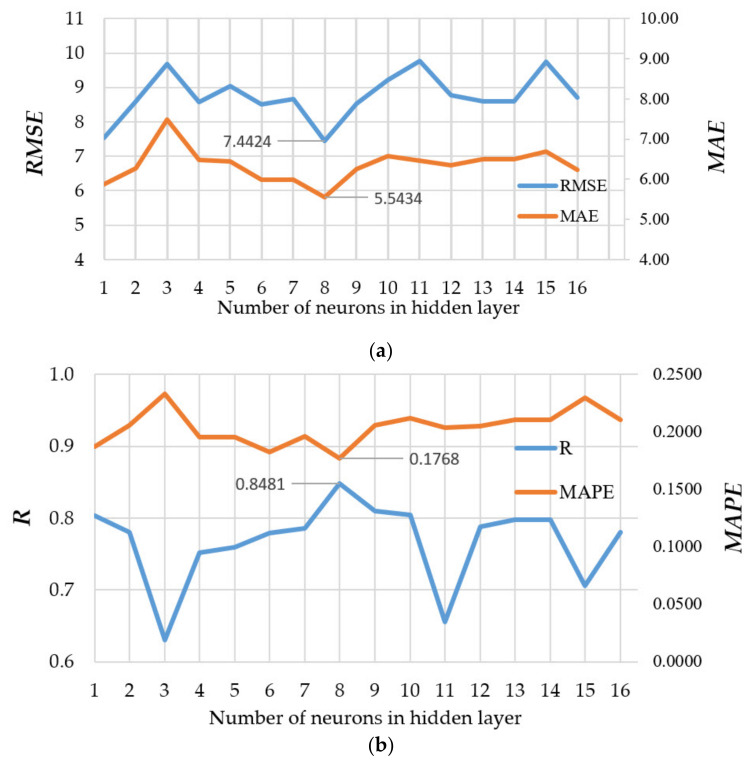
Comparison of performance measures using MLP-ANNs with different configurations: (**a**) *RMSE* and *MAE*, (**b**) *R* and *MAPE*.

Figure 7a shows the obtained performance using *RMSE* and *MAE* as absolute measures, while Figure 7b presents results using *R* and *MAPE* as relative measures. By analyzing models with different numbers of neurons in the hidden layer, it was concluded that the configuration with eight neurons in the hidden layer was optimal considering all four criteria.

In order to improve the generalization of the model, ensemble models were created. The use of base models of neural networks having up to 16 neurons in their hidden layer were analyzed, where each of the base models in the ensemble could have a different number of neurons in the hidden layer. The optimal base model in the current iteration was defined based on the minimum *RMSE* value of the 16 generated models in the current iteration. After that, the procedure continues until a total number of 100 base models of the ensemble were generated.

The bootstrap method formed a sample of the same size as the original sample. Since the evaluation used a ten-fold cross-validation procedure, sampling was performed within nine folds. The remaining fold was used to test the ensemble. The procedure was repeated 10 times so that the whole set of data was used for testing the ensemble, and the evaluation of the prediction of the ensemble was represented by the mean value of all base models in terms of the considered model performance.

**Figure 8:**
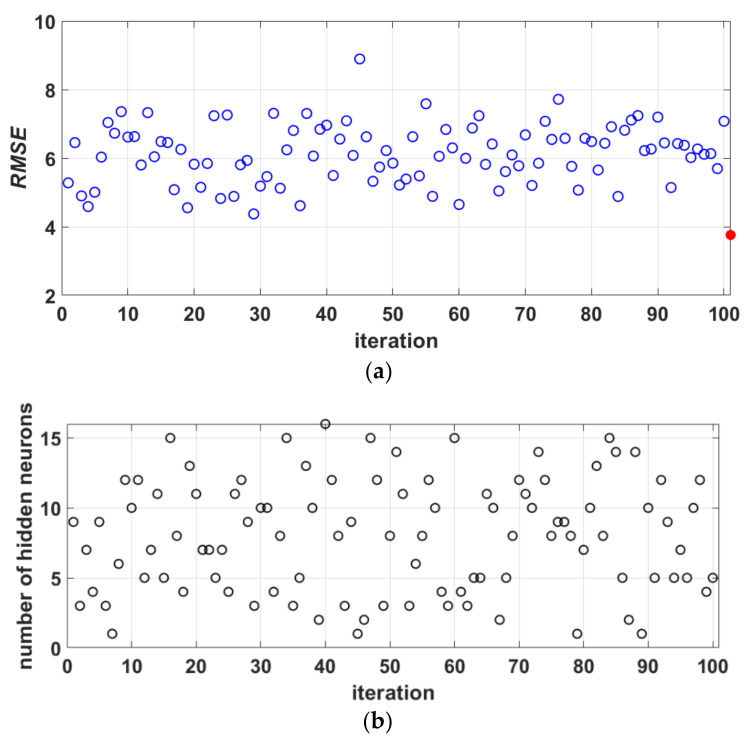
(**a**) *RMSE* value for each of the iterations and the corresponding architecture; (**b**) the optimal number of neurons in the hidden layer in each iteration.

In Figure 8, there is no trend in terms of accuracy to which an ANN model with a certain number of neurons in the hidden layer stands out. Figure 8 shows 100 ANN models generated on different samples obtained by bootstrap aggregation, where the optimal number of hidden layer neurons varies, as well as the achieved accuracy with respect to *RMSE* which is generally unsatisfactory in all models. The accuracy of individual ANN models varies mainly between 4 and 8. By including these individual models in the ensemble, although the complexity increases, the accuracy in terms of RMSE criteria becomes significantly higher (the red circle in Figure 8a represents the RMSE value for the ensemble). There is also a significant increase in accuracy with regard to other accuracy criteria.

**Figure 9:**
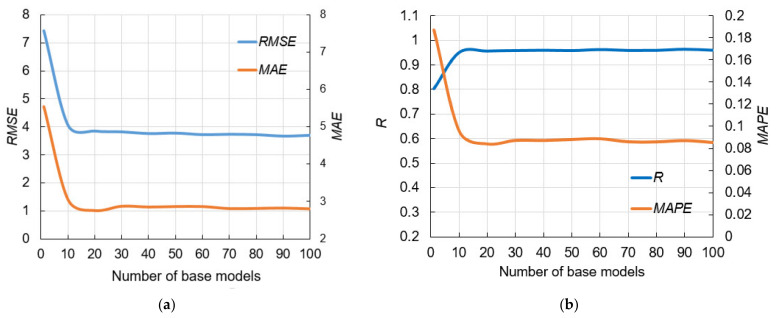
Comparison of performance measures using ensembles of MLP-ANNs with different number of base models: (**a**) *RMSE* and *MAE*, (**b**) *R* and *MAPE*.

**Figure 10:**
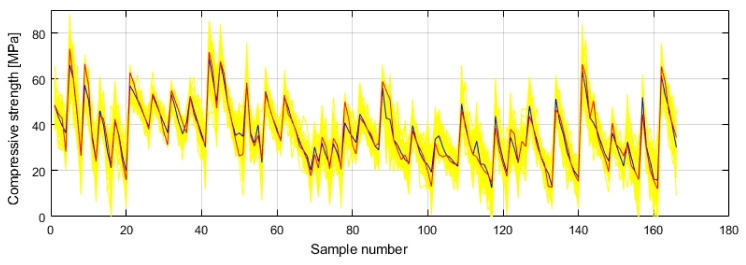
Prediction of individual neural networks (yellow color), ensemble model prediction (dark blue color) and target values (red color) of compressive strength.

**Figure 11:**
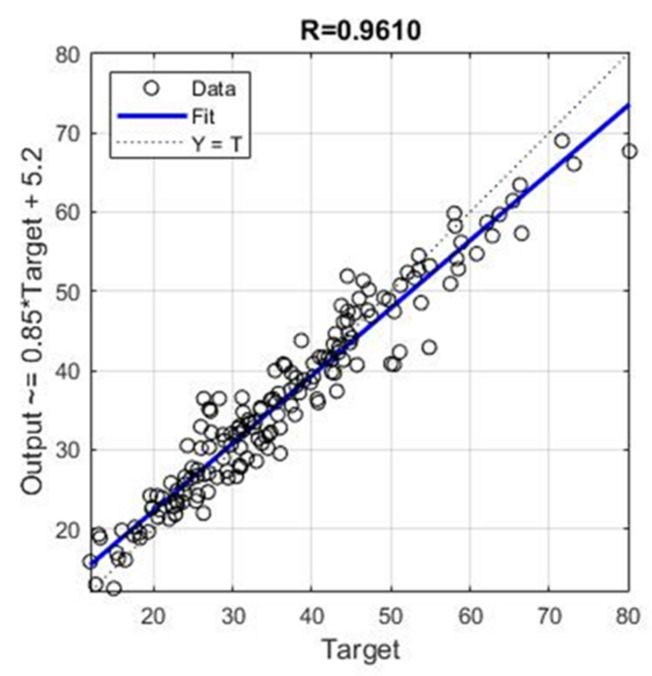
Regression plot for modelled and target values for optimal ensemble model.

It can be seen in Figure 9 and Figure 10 that the model of an ensemble composed of individual neural networks contributes to a significant improvement in generalization. Comparative values in terms of defined criteria for model evaluation for the optimal individual neural network model and ensemble model are shown in Table 4, and the regression plot of the modelled and target values for the ensemble model is shown in Figure 11.

The application of models based on decision trees was also analyzed. *MSE* values were used as a criterion in model training, and numerical values of *MSE* of the base models of the generated ensemble were presented cumulatively. The parameters of the optimal models were determined by the grid-search method.

The analysis was performed using the following methods:

1. Bagging method (TreeBagger),
2. RF method,
3. Boosted Trees method.

The data used for training (in bag data) in the TreeBagger model are extracted from the entire data set by sampling with replacement. Data that are not extracted from the whole set (out of bag data) represent test data. During the model building process, an out-of-bag error is calculated by finding the difference between the out-of-bag sample and the prediction for that same sample, and it is stored. The procedure is repeated for all trees within the ensemble.

During the implementation of the Bagging method, different values of model parameters were analyzed, as follows:

1. Number of generated trees B. Within this analysis, the maximum number of generated trees was limited to 500.
2. The minimum number of data or samples assigned to the leaf (min leaf size) within the tree. Values from 2 to 15 samples with a step size of 1 per tree leaf were considered.

**Figure 12:**
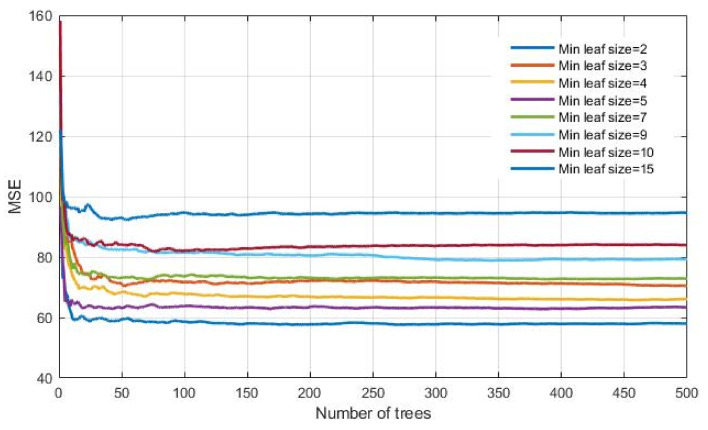
*MSE* vs. number of trees in the ensemble for different minimum leaf sizes using regression tree ensembles realized with bootstrap aggregation (bagging).

The lowest value of *MSE* (Figure 12) of the analyzed models has a model that has a minimum number of data per tree leaf 2, marked in darker blue. The saturation of the learning curve occurs after 269 trees in the ensemble.

**Figure 13:**
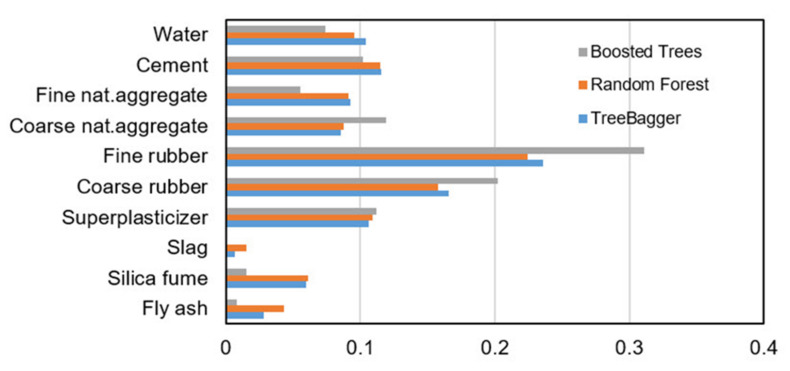
Significance of individual variables in the ensemble model when applying the Bagging method.

In order to determine the significance of the j-th variable, it is necessary that after training the model, the values of the j-th variable be permuted within the training data and that the out of bag error for such permutated data be recalculated. The significance of the variable (Figure 13) is determined by calculating the mean value of the difference before and after permutation for all trees within the ensemble. This value is then divided by the standard deviation of these differences. The variable for which a higher value was obtained in relation to the others is ranked as more significant in relation to the variables in which smaller values were obtained [36].

**Figure 14:**
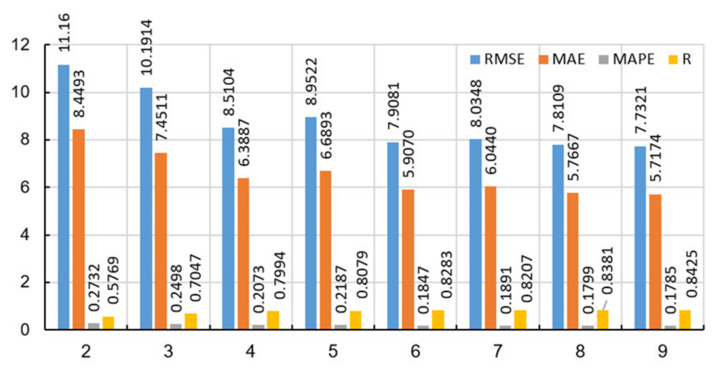
Influence of the number of variables on which is performed splitting on the accuracy of the model in the RF method.

During the implementation of the RF method, different values of the adaptive parameters of the model were analyzed, as follows:

1. Number of generated trees B. Within this analysis, the maximum number of generated trees was limited to 500.
2. The number of variables that are used for splitting in the tree. In the paper Random Forests by L. Breiman [38], it is recommended that the subset m of variables on which splitting is performed is p/3 of the predictor. In this paper, the values of m from 2 to 9 (Figure 14) are examined.
3. The minimum number of data or samples assigned to a leaf (min leaf size) within a tree. Values from 2 to 10 samples per tree leaf were considered.

The application of a narrowed set of splitting variables in this case did not yield results. Figure 14 shows the values of the accuracy criteria of the ensemble of 500 basic models, from which one can see the tendency to increase the number of variables upon which the splitting is performed to increase the accuracy of the model in terms of all defined criteria. When 2 variables, upon which the potential splitting of the tree is considered, are randomly selected from a set of 10 input variables, the model of least accuracy is obtained (*RMSE* = 11.16, *MAE* = 8.4493, *MAPE*/100 = 0.2732, *R* = 0.5769). On the other hand, randomly selecting a large number of variables for potential splitting increases the accuracy of the model and is greatest when using a set of 9 randomly selected variables from a set of 10 input variables (*RMSE* = 7.7321, *MAE* = 5.7174, *MAPE*/100 = 0.1785, *R* = 0.8425). An analysis that takes into account all 10 input variables when creating trees of the ensemble has already been done with the TreeBagger model.

The analysis showed greater accuracy in tree models in which the number of data per leaf is equal to 2. The relevance of individual variables for RF model is shown in Figure 13.

With Boosting Trees method, the following model parameters were considered:

1. Number of generated trees B. With the Gradient Boosting method, there is a possibility of overtraining the model when forming too many trees. Due to the large number of analyzed models in the research, the number of base models within the ensemble was limited to a maximum of 100.
2. Learning rate λ. This parameter determines the training speed of the model. The paper investigates a number of values, as follows: 0.001; 0.01; 0.1; 0.25; 0.5; 0.75 and 1.0.
3. Number of splits in the tree d. Models of trees with a maximum number of splits of 2^0^ = 1, 2^1^, 2^2^, 2^3^, 2^4^, 2^5^, 2^6^, 2^7^ = 128 were generated.

**Figure 15:**
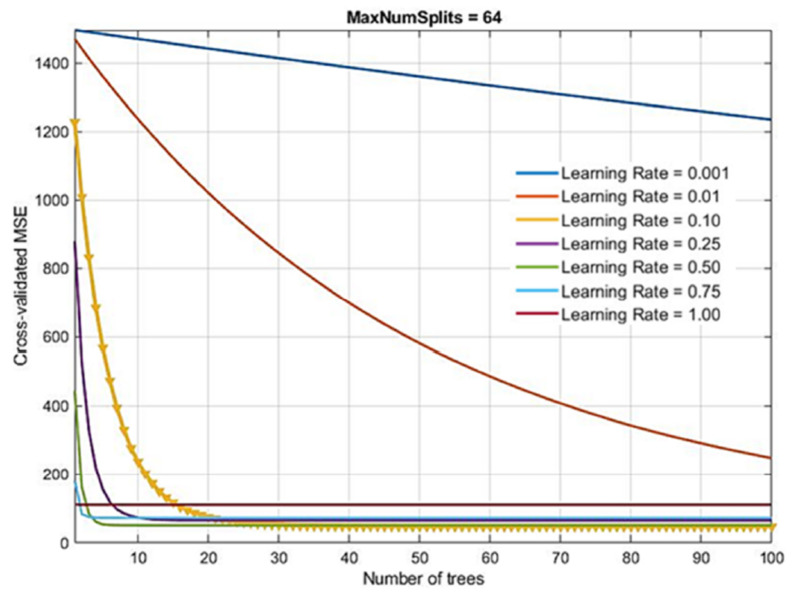
Dependence of *MSE* values on learning rate and number of base models in Boosted Trees method.

The optimal model obtained (marked in yellow in the Figure 15) had 100 generated trees, a reduction parameter value of 0.10 and a maximum number of splits of 64. As in other tree-based models, the relevance of individual model variables is determined and shown in Figure 13. A comparison of all tree-based models (Bagging, RF and Boosted Trees methods) is given in Table 5.

**Figure 16:**
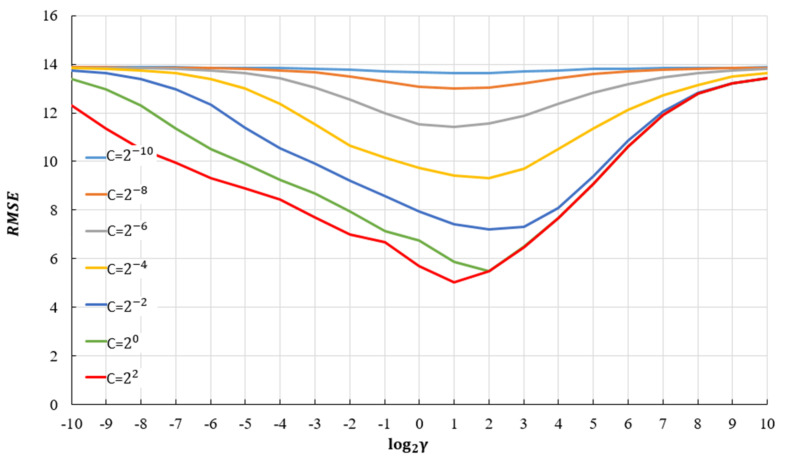
*RMSE* vs. hyperparameters C and γ for ε = 2^−6^ using SVR with RBF kernel in rough grid search.

In order to obtain a good regression model using the support vector method, it is necessary to select the appropriate kernel function. For the selected kernel functions, it is necessary to determine their parameters, as well as the value of the penalty parameter C (Figure 16).

In this paper, therefore, the use of several different kernel functions is investigated in order to find the best one. The use of SVR models with linear, RBF and sigmoid kernel was analyzed. Normalization, by which all input data were transformed into the range (0, 1), was done before training and testing the model. The optimal model was determined using the grid search algorithm for all kernels (C = 4.22195 and ε = 0.105765 for the linear kernel; C = 7.67645; ε = 0.0230915; γ = 1.89915 for the RBF kernel; C = 1113.70875; ε = 0.0920525; γ = 0.000945345 for sigmoid kernel).

Comparative analysis of different SVR models shows that the models have different accuracy depending on the adopted criteria depending on the kernel function. Models with linear and sigmoid kernels have similar accuracy according to different criteria. The model with the RBF kernel function (Table 6) has significantly higher accuracy with respect to all criterion functions.

During the development of the Gaussian process model, covariance functions that have one length scale parameter for all input variables (exponential, square-exponential, Matern 3/2, Matern 5/2) and rational quadratic covariance function as well as their equivalent ARD covariance functions that have a separate length scale for each input variable were considered. Standardization procedure was performed using Z-score, i.e., the data is transformed to have a mean value of zero and a variance equal to one. Models with constant base functions were analyzed.

Parameter values are determined (Table 7 and Table 8) by maximizing the log marginal probability. By using ARD covariance functions, it is possible to see the relevance of individual variables or predictors in the model (Automatic Relevance Determination-ARD). Higher parameter values for ARD covariance functions indicate less relevance of a particular variable to which they refer.

According to the three defined criteria, the *RMSE*, *MAE* and *R*, the model with ARD Matern 3/2 covariance function (Table 9) can be considered optimal, while according to the *MAPE* criterion it is second in accuracy with a difference of 0.0011 compared to the first ranked model according to that criterion.

**Figure 17:**
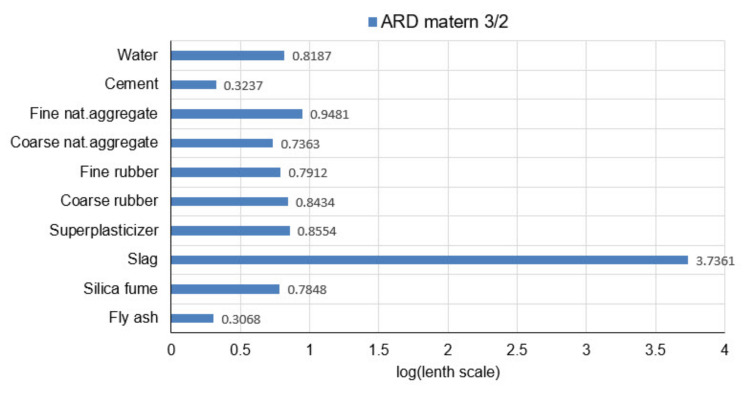
Variable selection using ARD Matern 3/2 covariance function.

The analysis of the relevance of the variable models will be performed based on the parameters of the covariance function on the model with the ARD Matern 3/2 function as the most accurate model. The values of the distance scale parameters are shown in the logarithmic scale (logarithms with base 10) in Figure 17.

It can be seen (Figure 17) that in the optimal model with ARD Matern 3/2 function the input variables 2 (cement) and 10 (fly ash) have the greatest relevance and the greatest impact on the model’s accuracy. Variables 1 (water), 3 (fine natural aggregate), 4 (coarse natural aggregate), 5 (fine rubber), 6 (coarse rubber), 7 (superplasticizer) and 9 (silica fume) have similar relevance. Input variable 8 (slag) has the least relevance and the most negligible impact on the model’s accuracy.

**Figure 18:**
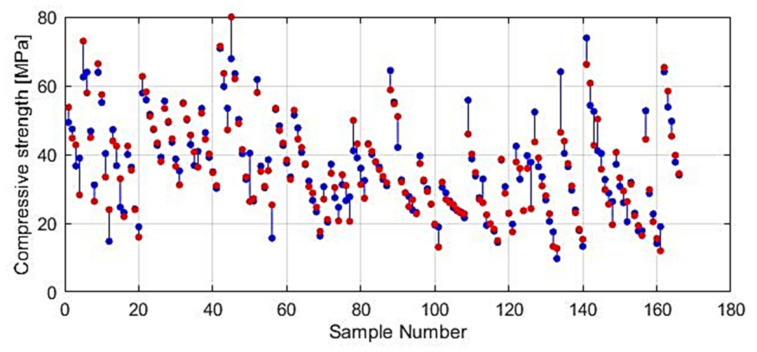
Modelled and target values for reduced GPR ARD Matern 3/2 model.

**Figure 19:**
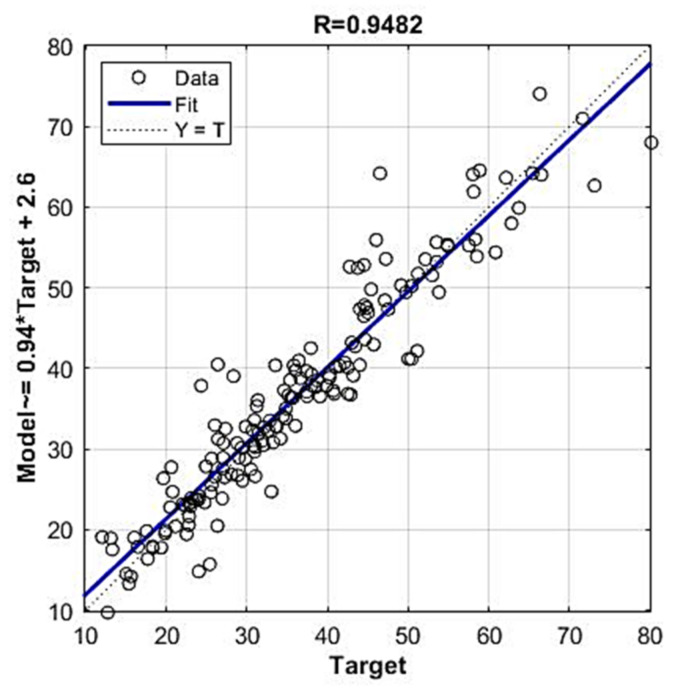
Regression plot of modelled and target values of compressive strength for reduced GPR ARD Matern 32 model.

In further analysis, the models that used the most relevant variables were considered due to the possibility that the presence of irrelevant variables may reduce the accuracy of the model. By using a narrowed set of variables in certain cases, it is possible to obtain a model (Figure 18 and Figure 19) of the same or higher accuracy. Additionally, in this way, the complexity of the model is reduced, and the model training process is accelerated.

In the further analysis, a comparison was made (Table 10) of the two models, as follows:

1. Model where variable 8 is excluded as less relevant (slag),
2. A model that includes all variables.

The application of individual models of neural networks gave models with unsatisfactory accuracy in terms of all criteria. For this reason, the use of neural network ensembles was considered. A limit is defined in terms of the maximum number of hidden layer neurons, while the number of inputs and outputs defines the number of input and output layer neurons. The analysis showed that the application of ensembles gives models of significantly higher accuracy than the optimal individual model of the neural network with the architecture 10-8-1. The value of the ensemble correlation coefficient was increased to the value 0.9610, which is a satisfactory value. The values of *RMSE*, *MAE* and *MAPE* are approximately halved in relation to the individual optimal neural network model. With an ensemble of 40 base models, there is convergence in terms of defined criteria, and a further increase in the complexity of the model does not lead to an increase in accuracy.

A comparative analysis of all tree-based models shows that the model obtained by applying Boosted Trees has the best accuracy, while models based on the Bagging and Random Forests methods showed lower and similar values in terms of all criteria. All models based on regression trees can determine the relevance of individual input variables in the model. As the least relevant input variable, all models identified the variable denoting slag. In the event of a significant expansion of the database, the complexity of the method could be analyzed, whereby the models in the Bagging and Random Forests methods can be processed in parallel, which is not the case with the Boosted Trees method, where models are trained sequentially within the ensemble. In addition, it should be pointed out that a significant number of basic models are needed to saturate the learning curve.

Comparative analysis of different SVR models shows that the models, depending on the kernel function, have different accuracies depending on the adopted criteria. The use of the RBF kernel function, in this case, gave satisfactory results, while the use of linear and sigmoid kernels gave significantly worse results. Models with linear and sigmoid kernel function have values tht are almost twice as bad in terms of the three criteria RMSE, MAE, and MAPE. The value of the correlation coefficient R is significantly lower than the RBF model. Training models with the RBF function are relatively simple, but these models do not provide direct insight into the relevance of individual variables in the model.

GPR models that do not use scale parameters of different lenghts for individual input variables in the considered problem in most cases have worse criterion values than models that use different length scale parameters (ARD models). The best model is a model with ARD Matern 3/2 covariance function. The values of the length scale parameters for the optimal model can be used to assess the relevance of the predictor or variable in the model. This model singles out fly ash and cement as the most significant variables. Slightly smaller but of similar relevance are the variables representing coarse natural aggregate, silica fume and fine rubber. The variables coarse rubber and superplasticizer represent the next group of variables that have similar and lower relevance than the previous. The least relevant are the variables fine natural aggregates and slag.

In further analysis, GPR models that used the most relevant variables were considered due to the possibility that the presence of irrelevant variables may reduce the accuracy of the model accuracy. It has been shown that by eliminating the variable of smaller significance, which in this case represents the variable Slag, a model of higher accuracy is obtained. Additionally, in this way, the complexity of the model is reduced, and the model training process is accelerated.

## 6. Conclusions

This paper gives a comprehensive overview of machine learning methods that can be used for estimating SCRC compressive strength, including MLP-ANN, ensembles of MLP-ANNs, regression tree ensembles (random forests, boosted and bagged regression trees), SVR and GPR, with different covariance functions.

As a basis for the development of the forecast model, a database containing a total of 166 samples of SCRC was obtained from various experimental studies.

Ensembles of neural networks and GPR models with ARD covariance function stood out as the models of the highest accuracy. Other analyzed models are more complex, and optimization of their parameters requires significant search efforts, but they are still less accurate.

The following values of accuracy criteria were obtained for the ensemble of neural networks: *RMSE* = 3.6888, *MAE* = 2.8099, *MAPE* = 0.0854 and *R* = 0.9610, while the GPR model with Matern 3/2 covariance function had values of *RMSE* = 4.3934, *MAE* = 3.0583, *MAPE* = 0.0942 and *R* = 0.9482.

The application of an ensemble of neural networks has the greatest complexity, where satisfactory accuracy is achieved only with the formation of an ensemble with a significant number of basic models. Neural network ensembles do not allow direct consideration of the relevance of individual input variables.

The use of the GPR method gives models of satisfactory accuracy and, at the same time, significantly less complexity. The proposed model with the ARD Matern 3/2 covariance function enables the ranking of the influence of individual variables on the accuracy of the model. The complexity of this model is reduced, and the model training process is accelerated.

Based on the proposed models for the neural network ensemble and GPR models, a model application was developed in MATLAB and deposited on the Github website.

## Figures and Tables

**Table 1:** Algorithm used in modelling the compressive strength of rubberized concrete and SCRC.

Type of Concrete	Algorithm	Data Points	Authors	Reference
Rubberized concrete	ANN, fuzzy logic (FL)	36	Topçu et al.	[19]
Rubberized concrete	ANN, gene-expression programming (GEP)	70	Gesoglu et al.	[20]
Rubberized concrete	ANN	287	El-Khoja et al.	[21]
Rubberized concrete	ANN, k-nearest neighbor (KNN), regression trees (RT) and random forests (RF)	457	Hadzima-Nyarko et al.	[22]
Rubberized concrete	GPR, SVM	89	Gregori et al.	[23]
Rubberized concrete	ANN	129	Dat et al.	[24]
Rubberized concrete	ANN	353	Huang et al.	[25]
Rubberized concrete	RF	138	Sun et al.	[26]
Rubberized concrete	ANN	122	Bachir et al.	[27]
Rubberized concrete	Self-adaptive fuzzy least squares support vector machines inference model (SFLSIM)	70	Cheng and Hoang	[28]
SCRC	Gaussian process regression (GPR)	144	Hadzima-Nyarko et al.	[2]
SCRC	Beetle antennae search (BAS)-algorithm-based random forest (RF)	131	Zhang et al.	[29]

**Table 2:** Average, minimum and maximum values of input and output variables used for modelling.

Variable	Average Value	Minimum Value	Maximum Value
Water (kg/m^3^)	197.15	170.00	246.00
Cement (kg/m^3^)	402.39	180.00	550.00
Fine nat. aggregate (kg/m^3^)	764.32	375.20	1192.00
Coarse nat. aggregate (kg/m^3^)	744.45	364.00	898.00
Fine rubber (kg/m^3^)	41.33	0	198.73
Coarse rubber (kg/m^3^)	18.20	0	355.80
Superplasticizer (kg/m^3^)	4.71	1.06	22.00
Slag (kg/m^3^)	23.08	0	175.00
Silica fume (kg/m^3^)	9.97	0	60.00
Fly ash (kg/m^3^)	74.26	0	330.00
SCRC compr. Strength (MPa)	74.26	0	330.00

**Table 3:** Statistical analysis of the properties of the database of SCRC specimens.

Year	Author(s)	Ref.	Type of Aggregate Replaced by Rubber	No. of Specimens	SCM ^1^	No. of SCM
2008	Turatsinze and Garros	[57]	Coarse	5	-	-
2010	Guneyisi	[58]	Coarse	16	FA ^2^	12
2012	Emiroglu et al.	[50]	Coarse	3	S ^3^	3
2012	Long et al.	[59]	Fine	6	SF ^4^ + FA	6
2013	Ganesan et al.	[60]	Fine	3	FA	3
2013	Yung et al.	[51]	Fine	5	S + FA	5
2014	Li et al.	[52]	Fine	7	SF + FA	7
2015	Ismail et al.	[61]	Fine	5	-	-
2015	Khalil et al.	[53]	Fine	5	-	-
2015	Mishra and Panda	[62]	Coarse	5	-	-
2016	Guneyisi et al.	[63]	Fine and Coarse	21	FA	21
2017	Ismail and Hassan	[56]	Fine	16	FA	3
					S	3
2016	Padhi and Panda	[64]	Fine	4	-	-
2016	Yu	[54]	Fine	6	FA	6
2016	Zaoiai et al.	[55]	Fine and Coarse	5	-	-
2017	Bideci et al.	[65]	Coarse	4	S	4
2018	AbdelAleem and Hassan	[66]	Fine	12	S	1
					FA	1
					SF	10
2018	Aslani et al.	[67]	Fine and Coarse	13	S + FA + SF	13
2018	Hamza et al.	[68]	Fine	4	-	-
2019	Yang et al.	[69]	Fine	4	SF + FA	4
2020	Bušić et al.	[70]	Fine	17	SF	10
-	-	-	Total	166	-	-

^1^ Supplementary cementing material; ^2^ FA—Fly Ash; ^3^ S—Slag; ^4^ SF—Silica Fume.

**Table 4:** Comparison of the optimal individual neural network and ensemble model.

Model	*RMSE*	MAE	*MAPE/*100	R
NN-10-8-1 *	7.4424	5.5434	0.1768	0.8481
Ensemble	3.6888	2.8099	0.0854	0.9610

* NN-10-8-1 is a model of an artificial neural network that is optimal according to four defined criteria RMSE, MAE, MAPE and R (Figure 7) that has 10 neurons in the input layer, 8 neurons in the hidden layer, and 1 neuron in the output layer.

**Table 5:** Comparative analysis of results in Bagging, RF and Boosted Trees methods.

Method	*RMSE*	*MAE*	*MAPE/*100	*R*
TreeBager	8.1890	6.0546	0.1881	0.8214
RF	7.7321	5.7174	0.1785	0.8425
Boosted Trees	**7.4821**	**5.4248**	**0.1573**	**0.8432**

**Table 6:** Comparative analysis of results using linear, RBF and sigmoid kernel in SVR method.

Model	*RMSE*	*MAE*	*MAPE/*100	*R*
Lin. kernel	8.7154	6.6468	0.2105	0.7751
RBF kernel	**4.9646**	**3.5352**	**0.1171**	**0.9332**
Sig. kernel	8.7104	6.6094	0.2073	0.7718

**Table 7:** Parameters of GPR ARD model covariance functions.

GP Model Covariance Function	Covariance Function Parameters			
Exponential	$k\left(\left(x_{i},x_{j}|\Theta \right)\right)=\sigma_{f}^{2}exp\left[-\frac{1}{2}\frac{r}{\;\sigma_{l}{}^{2}}\right]$			
	$\sigma_{l}=$51.7642		$\sigma_{f}=$44.6486	
Squared Exponential	$k\left(\left(x_{i},x_{j}|\Theta \right)\right)=\sigma_{f}^{2}exp\left[-\frac{1}{2}\frac{{\left(x_{i}-x_{j}\right)}^{T}\left(x_{i}-x_{j}\right)}{\;\sigma_{l}{}^{2}}\right]$			
	$\sigma_{l}=$1.9621		$\sigma_{f}=$21.4682	
Matern 3/2	$k\left(\left(x_{i},x_{j}|\Theta \right)\right)=\sigma_{f}^{2}\left(1+\frac{\sqrt{3}r}{\sigma_{l}}\right)exp\left[-\frac{\sqrt{3}r}{\sigma_{l}}\right]$			
	$\sigma_{l}=$4.4183		$\sigma_{f}=27.5201$	
Matern 5/2	$k\left(\left(x_{i},x_{j}|\Theta \right)\right)=\sigma_{f}^{2}\left(1+\frac{\sqrt{5}r}{\sigma_{l}}+\frac{5r^{2}}{3\sigma_{l}{}^{2}}\right)exp\left[-\frac{\sqrt{5}r}{\sigma_{l}}\right]$			
	$\sigma_{l}=$2.8760		$\sigma_{f}=23.1271$	
Rational Quadratic	$k\left(\left(x_{i},x_{j}|\Theta \right)\right)=\sigma_{f}^{2}{\left(1+\frac{r^{2}}{2a\sigma_{l}{}^{2}}\right)}^{-\alpha }$			
	$\sigma_{l}=$2.8568	a=0.3520		$\sigma_{f}=$28.5379

where $r=\sqrt{{\left(x_{i}-x_{j}\right)}^{T}\left(x_{i}-x_{j}\right)}$.

**Table 8:** Parameters of GPR ARD model covariance functions.

Covariance Function Parameters									
$\sigma_{1}$	$\sigma_{2}$	$\sigma_{3}$	$\sigma_{4}$	$\sigma_{5}$	$\sigma_{6}$	$\sigma_{7}$	$\sigma_{8}$	$\sigma_{9}$	$\sigma_{10}$
ARD Exponential: $k\left(\left(x_{i},x_{j}|\Theta \right)\right)=\sigma_{f}^{2}\exp\left(-r\right)$;$\;\sigma_{F}$=61.7884; $r=\sqrt{\overset{d}{\underset{m=1}{\sum }}\frac{{\left(x_{im}-x_{jm}\right)}^{2}}{\;\sigma_{m}{}^{2}}}$									
136.5920	34.7143	149.6719	87.1631	160.3637	137.0799	174.2748	380.2212	84.5739	42.4424
ARD Squared exponential: $k\left(\left(x_{i},x_{j}|\Theta \right)\right)=\sigma_{f}^{2}exp\left[-\frac{1}{2}\overset{d}{\underset{m=1}{\sum }}\frac{{\left(x_{im}-x_{jm}\right)}^{2}}{\;\sigma_{m}{}^{2}}\right];$$\sigma_{f}$=24.1382									
2.7611	0.8842	4.3944	3.1684	2.3792	2.6952	1.8852	4125.1386	5.6892	0.7528
ARD Matern 3/2: $k\left(\left(x_{i},x_{j}|\Theta \right)\right)=\sigma_{f}^{2}\left(1+\sqrt{3}r\right)exp\left[-\sqrt{3}r\right];$$\sigma_{f}$=32.6244									
6.5869	2.1073	8.8734	5.4483	6.1835	6.9731	7.1678	5445.6855	6.0925	2.0265
ARD Matern 5/2: $k\left(\left(x_{i},x_{j}|\Theta \right)\right)=\sigma_{f}^{2}\left(1+\sqrt{5}r+\frac{5r^{2}}{3}\right)exp\left[-\sqrt{5}r\right];$$\sigma_{f}$=26.5499									
3.8496	1.2738	6.9524	4.5500	3.5908	3.9290	2.6596	2276.2435	5.8061	1.3211
ARD Rational quadratic: $k\left(\left(x_{i},x_{j}|\Theta \right)\right)=\sigma_{f}^{2}{\left(1+\frac{1}{2\alpha }\overset{d}{\underset{m=1}{\sum }}\frac{{\left(x_{im}-x_{jm}\right)}^{2}}{\;\sigma_{m}{}^{2}}\right)}^{-\alpha };\;\alpha$=0.3332; $\;\sigma_{f}$=61.7884									
3.6879	1.3166	7.3725	4.6652	3.7047	3.9596	2.6138	4383.7159	5.4661	1.4843

where $r=\sqrt{\overset{d}{\underset{m=1}{\sum }}\frac{{\left(x_{im}-x_{jm}\right)}^{2}}{\;\sigma_{m}{}^{2}}}$.

**Table 9:** Comparative analysis of results of GPR with various covariance functions.

GP Model Covariance Function	*RMSE*	*MAE*	*MAPE/*100	*R*
Exponential	5.0574	3.5064	0.1038	0.9316
ARD-Exponential	4.6120	3.1634	**0.0947**	0.9427
Squared Exponential	5.0447	3.4686	0.1133	0.9300
ARD-Squared Exponential	4.9670	3.4076	0.1101	0.9334
Matern 3/2	4.7244	3.2487	0.1021	0.9386
ARD-Matern 3/2	**4.4341**	**3.1022**	0.0958	**0.9474**
Matern 5/2	4.8275	3.3133	0.1061	0.9360
ARD-Matern 5/2	4.6527	3.2691	0.1037	0.9424
Rational Quadratic	4.6467	3.2022	0.0997	09407
ARD Rational quadratic	4.5937	3.1894	0.1006	0.9435

**Table 10:** Comparative analysis of GPR models with different sets of input variables.

Model	$x_{1}$	$x_{2}$	$x_{3}$	$x_{4}$	$x_{5}$	$x_{6}$	$x_{7}$	$x_{8}$	$x_{9}$	$x_{10}$	RMSE	MAE	MAPE/100	R
1.	1	1	1	1	1	1	1	0	1	1	4.3934	3.0583	0.0942	0.9482
2.	1	1	1	1	1	1	1	1	1	1	4.4341	3.1022	0.0958	0.9474

## Data Availability

Not applicable.
